# Insights into tertiary lymphoid structures in the solid tumor microenvironment: anti-tumor mechanism, functional regulation, and immunotherapeutic strategies

**DOI:** 10.20892/j.issn.2095-3941.2021.0029

**Published:** 2021-09-24

**Authors:** Hua Zhao, Hao Wang, Qiuru Zhou, Xiubao Ren

**Affiliations:** 1 Department of Immunology, Tianjin Medical University Cancer Institute and Hospital, National Clinical Research Center for Cancer, Key Laboratory of Cancer Prevention and Therapy, Tianjin, Tianjin’s Clinical Research Center for Cancer, Key Laboratory of Cancer Immunology and Biotherapy, Tianjin 300060, China; 2 Department of Biotherapy, Tianjin Medical University Cancer Institute and Hospital, National Clinical Research Center for Cancer, Key Laboratory of Cancer Prevention and Therapy, Tianjin, Tianjin’s Clinical Research Center for Cancer, Key Laboratory of Cancer Immunology and Biotherapy, Tianjin 300060, China

**Keywords:** Tertiary lymphoid structure, tumor microenvironment, immunotherapy, B cells, T cells

## Abstract

Tertiary lymphoid structures (TLSs) are ectopic immune cell aggregations that develop in peripheral tissues in response to a wide range of chronic inflammatory conditions, including infection, autoimmune disease, and cancer. In the tumor microenvironment (TME), the structures of TLSs, including B-cell- and T-cell-enriched areas indicate that the TLSs might be the local site during the initiation and maintenance of humoral and cellular immune responses against cancers. Numerous studies have evaluated the expression of TLSs in different cancer patients and their association with prognoses of cancer patients. It was shown that well-developed TLSs characterized by mature B cells synthesized tumor specific antibodies, which were considered as specific markers for a good prognosis. However, there are still some immunosuppressive factors existing in the TLSs that may affect anti-tumor responses. These factors include dysfunctional B cells, regulatory T cells, and T follicular regulatory cells. The complexity and heterogeneity of the TLS composition may affect the function and activity of TLSs; it is therefore essential to fully understand the function and influencing factors in TLSs. It has been reported that checkpoint inhibitors and vaccines are currently being developed to reprogram the TME by establishing mature TLSs to improve cancer immunotherapies. In this review, we focused on recent advances in TLSs in human solid tumors, including structural characteristics and classes, antitumor mechanisms, immunosuppressive factors, and TLS-based therapeutic approaches.

## Introduction

The tumor microenvironment (TME) comprises a mass of heterogeneous cell types, including endothelial, fibroblast, and immune cells, which are surrounded by tumor cells and nourished by a vascular network. The TME not only plays a pivotal role during tumor initiation, progression, and metastasis, but also plays important roles in the efficacies of tumor treatments^1,2^. Among them, the immune cells play an important role in antitumor responses. The infiltration of activated immune cells is generally positively correlated with the prognoses of cancer patients^3,4^. During study of the TME^5^, researchers found immune cell aggregations that frequently developed in non-lymphoid tissues of pathological sites, which also could be found in chronic infections^6^, autoimmune diseases^7^, and allograft rejections^8^ because of long-term chronic inflammatory signal stimulation. Tertiary lymphoid structures (TLSs), also known as tertiary lymphoid organs or ectopic lymphoid organs, are one of the major sources of tumor infiltrating lymphocytes (TILs) and have exhibited important roles in antitumor immunity^9^.

A previous study^10^ evaluated the association of immune cell infiltration in TLSs with the immune checkpoint inhibition (ICI) therapy response in metastatic melanoma patients. It was reported that co-occurrence of tumor-associated CD8^+^T and CD20^+^B cells associated with TLSs had a significant association with improved survival, independent of other clinical variables. Furthermore, B cell-rich tumors were accompanied by increased levels of TCF7^+^ naive and/or memory T cells^10^. In a study of sarcomas (*n* = 213), the results showed that high densities of B cells and TLSs were associated with the highest response to ICI. Importantly, only a B cell signature was significantly associated with an extended overall survival (OS), which indicated an important antitumor function of antibodies^11^. Another neoadjuvant ICI trial in patients with melanomas and renal cell carcinomas also showed that B cell signatures within TLSs were enriched in the tumors of patients who responded to treatment, when compared with those from non-responding patients^12^. The findings from three independent studies indicated that B cells and TLSs were key determinants of responses to ICI^10–12^, suggesting the TLS is important in the antitumor immune response and is vital to fully understanding the functions and regulatory factors of TLSs in antitumor immunity.

## The composition of TLSs

TLSs reflect lymphoid neogenesis in peripheral nonlymphatic tissues in response to persistent chronic inflammatory stimulations. The TLSs are similar to secondary lymphoid organs (SLOs), especially lymph nodes, although there is no capsule surrounding the structure, which is different from SLOs. The cellular architecture of well-developed TLSs is characterized by B cell-enriched zones, which include B cell follicles organized around a network of follicular dendritic cells (FDC) and follicular helper T cells (Tfh), T cell-enriched zones organized around mature dendritic cells (DCs), specialized vessels known as high endothelial venules (HEVs) with lymphocyte extravasation, and lymphatic vessels for collection of antigens and immune cells from tissues as well as for the release of cells into the blood (**Table 1**)^13–17^.

**Table 1:** The immune characteristics of tertiary lymphoid structures in cancers

	Characteristic detecting markers	Subpopulation markers	Prognoses	Reference
**B cell zone**	CD20^+^B cells	Naïve B cells: CD19^+^CD20^+^IgD^+^	No evaluation	^13,17,18^
		Memory B cells: CD19^+^CD20^+^CD27^+^	No evaluation	^13,17,18^
		PCs: CD20^−^CD27^+^CD38^+^CD138^+^	Favorable	^13,19,20^
		Plasma blasts: CD20^−^CD27^+^ CD38^+^	Favorable	^13,19,20^
		FDC: CD21^+^, CD35^+^, CD23^+^, all the markers also expressed on B cells	Favorable	^13,19,20^
		Tfh: CD4^+^CXCR5^+^Bcl6^+^	Favorable	^19,20,36^
		Tfr: CD4^+^CXCR5^+^foxp3^+^	No evaluation	^68,69^
		GC: Bcl-6^+^CD20^+^B cells, or Ki67^+^CD20^+^ B cells	Favorable	^12,13,18^
		Breg: IL-10^+^IgA^+^CD19^+^	Unfavorable	^57,58^
		Macrophage: CD68^+^	Unfavorable	^64^
**T cell zone**	CD3^+^T cells, or	Naïve T cell: CCR7^+^CD45RO^−^T cells	No evaluation	^12^
	CD4^+^T cells or	Memory T cells: CD45RO^+^CD45RA^−^T cells	Favorable	^12^
	CD8^+^T cells	Tregs: CD4^+^foxp3^+^T cells	Unfavorable	^13,61^
		CTL: CD8^+^ Granzyme B^+^ T cells	Favorable	^12,13,61^
		Helper T cells: T-bet^+^Th1	Favorable	^12,15^
		GATA3^+^ Th2	Unfavorable	^64^
		RORγt^+^ Th17	No evaluation	^64^
		DC: LAMP3^+^ or CD83^+^ or CD86^+^	Favorable	^12,13^
**Others:**				
HEV	PNAd	PNAd or MECA79	Favorable	^17^
Chemokines	CXCL13/CXCL12	CCL-2,3,4,5,8,18,19,21; CXCL-9, 10,11,12,13	Favorable	^16,17,18^

## TLS location and its prognostic impact on cancer patients

TLSs can localize to the invasive margin of tumor tissues, which are called peritumor TLSs, and/or the core of tumor tissues, which are called intratumor TLSs^18^. Numerous studies and reviews have described the association of peritumor TLSs with the prognoses of cancer patients, which showed that the density of peritumor TLSs was associated with improved prognoses independent of other clinical variables, and could be an effective prognostic biomarker for cancer patients^19–22^. However, there are a few studies that reported the opposite results regarding the relationship of the TLS position in the TME (peritumor or intratumor) with survival of cancer patients^20,22^. The results from a study by Sofopoulos et al.^19^, which described the TLSs in breast cancer, contradicted these findings; this group investigated the prognostic significance of peritumoral TLSs, either alone or jointly with intratumoral densities. Peritumor TLSs were defined as those within the area up to 5 mm from the infiltrating tumor border. A subgroup of distal TLSs was also defined as within the distal normal breast tissue. Patients with high densities of both peritumor and distal TLSs had the worst outcomes^19^. In another oral cancer-associated TLS study, immunohistochemistry was used to evaluate TLS expressions in surgical sections of 65 patients^18^. This study defined 5 grades according to distinct locations and densities of the TLSs: grade 0, patients having no TLSs; grade 1, patients with peritumor TLSs counts ranging from 1–4 and no intratumor TLSs; grade 2, patients with peritumor TLSs counts > 4 with no intratumor TLSs; grade 3, patients with intratumor TLSs counts from 1–4; and grade 4, patients with intratumor TLSs counts > 4. There was no criteria assigned to the peritumor TLSs in grades 3 and 4, although some specimens in grades 3 or 4 had peritumor TLSs. Patients with grade 3 plus 4 had significantly improved disease-free survival and OS, when compared with patients with grade 1 plus 2 and grade 0. There was no survival difference between patients in grade 1 plus 2 and grade 0.

## Maturation classification of TLSs and their effects on the prognoses of cancer patients

With increasing understanding of the heterogeneity of TLSs, the function and activity of cells inside the tumor may be shown to play more significant roles in the prognoses of patients. In an early study, Dieu-Nosjean et al.^15^ studied the presence and the correlation of tertiary lymphoid structures with clinical outcomes in early non-small cell lung cancer (NSCLC) patients. All patients underwent complete surgical resections of their tumors, including multilevel lymph node sampling or lymphadenectomy, but none of them received preoperative chemotherapy or radiotherapy. Patients with an Eastern Co-operative Oncology Group performance status < 1 were eligible. Because it was difficult to achieve quantitative counting of TLSs, DC-lysosome-associated membrane glycoprotein (DC-LAMP^+^), a marker of mature DCs that was found exclusively in the TLSs, was used as a specific marker of these structures. The mature DC/T cell clusters were often surrounded by CD20^+^B cell follicles characterized by the presence of both a CD21^+^FDC network and Ki67^+^-proliferating germinal center (GC)-B cells, and the density of mature DCs was highly associated with favorable clinical outcomes of cancer patients. The number of tumors infiltrating lymphocytes, including CD3^+^T cells, CD20^+^B cells, and especially CD4^+^ and T-bet^+^Th1 cells, was increased in tumor tissues in the absence of more mature DCs. A later study further investigated the role of follicular B cells in TLSs and the potential link with the local humoral immune response in NSCLC patients. Seventy-four untreated patients with early stage NSCLC and 122 patients with advanced stage NSCLC treated with neoadjuvant chemotherapy were enrolled in this study. It was reported that B cell areas of TLSs had an ongoing humoral immune response. The densities of CD20^+^ B cells, especially GC-B cells, represented a new favorable prognostic biomarker of cancer patients, suggesting the link between TLSs and a protective B cell-mediated immunity^23^.

In recent years, multiparameter immune-fluorescence technology has been widely used to characterize the tumor immune microenvironment, and to assess the degree of TLS maturation and differentiation into different maturation stages. Kroeger et al.^16^ assessed the co-localization patterns of TLSs in 30 patients with high grade serous ovarian cancer (HGSC) and classified them into 4 types based on size, cellular composition, and degree of organization. Type I was small (approximately 20–50 cells), compact, and composed of CD4^+^ and CD8^+^T cells, B cells, and occasional DCs. Type II was larger (100–1,000 cells) and composed of CD4^+^, CD8^+^ T, and CD20^+^ B cells; these aggregates showed a diffuse pattern and lacked discrete zones or follicles. Type III represented fully developed TLSs, which contained prominent B cell follicles with GC-like structures characterized by the expression of CD21^+^FDC, and discrete T cell zones with CD4^+^ and CD8^+^ T cells, DCs, and PNAd^+^ HEV-like vessels. Finally, type IV was composed of approximately 100–300 CD20^+^ B cells and FDC were organized into follicles with few CD4^+^ and CD8^+^ T cells, which did not contain clear T cell zones. In addition, the GC-B cells could be found in type III and type IV aggregates but not in type I and type II cells. TLS-associated GCs could serve as sites of prostate cancer differentiation. These TLSs were stained with CD38, CD138, and CD79a to distinguish GCs (which are CD20^−^ but co-express CD38, CD138, and cytosolic CD79a) from naïve and memory B cells (which are CD38^−^CD138^−^ but express membranous CD79a) and CD138^+^ tumor or stromal cells. Only the TLS type, including PCs, showed a positive association with increased survival in HGSC patients. Furthermore, Posch et al.^24^ analyzed the TLS numbers and degree of TLS maturation and their associations with effective antitumor immune surveillance in 109 patients with stage II/III nonmetastatic colorectal carcinoma. According to the increasing prevalence of FDCs and mature B cells, the maturations of TLSs were classified into 3 sequential stages as follows: 1) early TLSs (E-TLSs), composed of dense lymphocytic aggregates without FDCs; 2) primary follicle-like TLSs (PFL-TLSs), having FDCs but no GC reactions; and 3) secondary follicle-like TLSs (SFL-TLSs), having active GC reactions (**Table 2**). The data showed that patients with a low TLS density, a high proportion of E-TLSs, and low proportion of PFL-TLSs showed a tendency toward higher risk of recurrence. These findings were further confirmed in lung squamous cell carcinomas^25^. All these classifications were based on the maturation of B cells in the TLSs of the TME, which indicated that humoral immunity played an important role in the antitumor immune response.

**Table 2:** Maturity classifications of tertiary lymphoid structures

	Definition and cellular composition	Marker cell subsets
E-TLS	Dense lymphocytic aggregates without FDCs	CD4^+^ and CD8^+^T cells, CD20^+^B cells, and occasional DCs
PFL-TLS	Having more dense lymphocytic aggregates with FDCs but no GC reaction	CD4^+^ and CD8^+^T cells, CD20^+^B cells, and/or occasional DCs, and FDC
SFL-TLS	Having dense lymphocytic aggregates with FDCs and GC reaction	Bcl6^+^or Ki67^+^CD20^+^B cells, CD38^+^ and/or CD138^+^ CD19^+^B cells

## Antitumor mechanism of TLSs

### Humoral immunity

B cells are recognized as the main effector cells of TLS-associated humoral immune responses^23,24^. GCs are the microanatomical sites of B cell mutations and antibody affinity maturations. GC responses require generation (GC cell division), mutation, and immunoglobulin selection and maturation^26–28^. In tumor-associated TLSs, GC-B cells can express activation-induced cytidine deaminase, the critical enzyme for somatic hypermutation (SHM), class switch recombination (CSR), and conversion of immunoglobulin genes. GC-B cells were positive for the proliferation markers Ki67 and Bcl6, which indicated that SHM and CSR were activated, which were required for the generation of effector and memory B cells after B cell activation. CD138^+^ plasma cells (CD138^+^PC) could also be detected at the periphery of B follicles in TLSs, involving the stroma and fibrosis of tumors^13,14,18^. GC-B cells in NSCLC produced elevated levels of IgG and IgA, which displayed reactivities with tumor antigens (TAs) that have been described in many kinds of cancer types, such as anti-NY-ESO-1 and anti-XAGE-1b Abs^22^, although IgA may function as an important immunoglobulin in mucosal autoimmunity^29,30^. A panel of 34 tumor-associated antigens (TAAs) expressed in esophago-gastric adenocarcinomas (EACs) was identified based on public databases and The Cancer Genome Atlas (TCGA) datasets to analyze tumor-specific B cell responses. The results showed that 48.1% of patient serum contained tumor-specific IgG antibodies against ≥ 1 TAAs^31^. In HGSC metastases, B cells were located mainly in TLSs and had a memory phenotype. The IgG targeting TAs included IgG1 and IgG3^32^. These antibodies bound to the Fcγ receptor to trigger antibody-dependent cellular cytotoxicity and antibody-mediated phagocytosis, to mediate complement-based cytotoxicity^33–36^. It was reported that IgG1 and IgG3 efficiently triggered this classical complement route, but IgG2 and IgG4 were less efficient, or were efficient only under certain conditions for IgG2^37,38^. Shi et al.^39^ reported an atypical memory subset of IgD^−^IgG^+^CD27^−^CD38^−^ CD20^+^ B cells in human hepatocellular carcinomas, which killed tumor cells directly through the secretion of granzyme B and TRAIL. All of these results suggested that TLSs, centered on B cells, played critical roles in activating humoral immune responses, thereby influencing cancer prognoses.

The antitumor effects of B cells depend on complete activation and maturation of B cells. As in SLO, this process is completed the help of Tfh cells with high expression of CXCR5, inducible T cell co-stimulator (ICOS), programmed death 1 (PD-1), and B cell lymphoma 6 (Bcl6)^40,41^. CXCR5 is a chemokine receptor promoting B and T cell migrations to B cell follicles in response to CXCL13. Bcl6 is essential for the development and persistence of Tfh cells *in vivo* and promotes the expression of many Tfh cell-associated factors, including CXCR5, ICOS, PD-1, and CXCL13^40,41^. Human Tfh cells produce substantial amounts of CXCL13, which help recruit CXCR5^+^ B cells to follicles. The interactions of Tfh-B cells enhance Bcl6 expression and stabilize the phenotype of Tfh cells partly through ICOS-ICOSL interaction. Tfh cells stimulate B cell proliferation in GC and differentiate into plasma cells through IL-21 and CD40L signals. FDCs are retained in the GC and provide antigens to the B cells. In addition, Tfh cells also express elevated levels of PD-1, which weaken the maintenance of antigen-induced Tfh cells^27,42,43^. This effect may help eliminate excess Tfh cells and prevent the generation of low affinity antibodies^44,45^. Overall, these data indicated that Tfh help was a key limiting resource in GCs and that Tfh-mediated effector functions were critical to the development of humoral immunity.

### Cellular immunity

In addition to antibody-mediated humoral immunity, TLSs are important for cellular immunity, characterized by clusters of mature DCs and T cells. Behr et al.^46^ reported that in merkel cell carcinomas, 21 primary tumors were stained with immune cell markers including CD3, CD4, CD8, CD68, CD20, and S100. Tumor-infiltrating neutrophils, and TLSs and PD-L1 expressions, were analyzed and correlated with overall and recurrence free survival. The presence of TLSs in the TME significantly correlated with a favorable recurrence-free survival. In addition, the presence of TLSs significantly correlated with higher CD8/CD4 ratios in the tumor periphery, but not in the center of the tumor. It was reported that a high density of mature DCs in TLSs closely correlated a strong infiltration of CD62L^+^CD4^+^T, CD69^+^CD8^+^T, and CD62L^+^CD8^+^T cells and was predominantly of the effector-memory phenotype (CD45RA^−^CCR7^−^CD27^+or−^CD28^+or−^CD8^+^T cells) in NSCLC^15^. Moreover, several studies confirmed that mature DCs in TLSs were specifically associated with Th1 and CD8^+^ cytotoxic T cell responses and that these findings correlated with improved long-term survival^47–49^.

In addition, B cells can act as antigen-presenting cells with high expressions of the co-stimulatory molecules, CD86 and CD80, and in turn facilitate T cell responses^35^. It was reported that the polyclonal CD4^+^ T cell response was closely related to the antibody levels in serum^46,47^. In this study, 11 of 13 cancer patients with serum antibodies to NY-ESO-1 (New York esophageal squamous cell carcinoma 1) had polyclonal CD4^+^ T cell responses directed against various known and previously undescribed NY-ESO-1 epitopes. NY-ESO-1 is a well-known cancer-testis antigen (CTAs) with re-expression in numerous cancer types, which has the ability to elicit spontaneous humoral and cellular immune responses^50^. In contrast, none of the 18 patients with negative serum antibodies of NY-ESO-1 had detectable CD4^+^ T cell responses. It was also shown that 12-mer determinants of NY-ESO-1 eliciting a CD4^+^ T cell response were peptide 87–98 with promiscuous HLA class II presentation, peptide 108–119 restricted by HLA-DP4, and peptides 121–132 and 145–156, both shorter epitopes from previously described HLA-DR4 peptides, also presented by HLA-DR7^51^. This study indicated that the strong humoral response to NY-ESO-1 may greatly facilitate antigen uptake by APCs with the resulting priming and cross-presentation leading to and maintaining vigorous CD4^+^ and CD8^+^ T cell responses to NY-ESO-1^51^. CD4^+^ T cells are critical in mediating this protective immunity and are essential for the development of functional CD8^+^ T cell memory^52^. In gastric cancer patients, high infiltration of T-bet^+^ T cells, high numbers of CD20^+^ B cell follicles, and low infiltration of foxp3^+^ T cells were associated with better relapse-free survival while CD8^+^ and IL17^+^ T cell densities were not. There was a significant association of TLS density with B cell aggregates and T-bet^+^ cells^53^. Bruno and co-workers^54^ reported that B cells in the TME of NSCLC patients locally presented antigens to CD4^+^ TILs. The activated CD19^+^CD20^+^CD69^+^CD27^+^CD21^+^B cells were associated with an effector T cell response (IFNγ^+^ CD4^+^ TILs). Alternatively, exhausted B cells (CD19^+^CD20^+^CD69^+^CD27^−^CD21^−^) were associated with the regulatory T cell (Tregs) phenotype (FoxP3^+^ CD4^+^ TILs). Furthermore, CD70 and CD27 were expressed in both CD20^+^B cells and CD8^+^ T cells after the interaction. The mRNA expression levels of CCL21, CXCL13, PD-L1, perforin, and granzyme B in TLSs were significantly higher than in non-TLSs. Therefore, B cells in TLSs might function as antigen-presenting cells, which has been associated with the induction of cytotoxic T cells^55^.

## Immunosuppressive factors in TLSs

**Figure 1:**
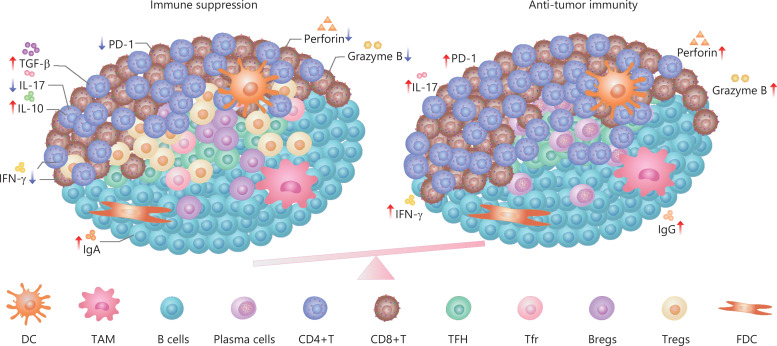
The immune regulation in TLSs.

Some immunosuppressive factors exist in TLSs (**Figure 1**). First, TLS-B cells can also secrete antigen-specific IgA in addition to IgG^56^. It was reported that a high intratumoral proportion of the IgA isotype was associated with a negative prognosis in the KRAS-mutant subtype of lung adenocarcinomas^57^. In prostate tumor-bearing mice, IgA^+^ B cells induce CD8^+^ cell exhaustion and suppress antitumor CTL responses through PD-L1 and IL-10, whose appearance depends on TGFβ-receptor (TGFβR) signaling^58^. It was reported that IL-10-expressing IgA^+^ cells were most abundant in patients with resistant and metastatic PCs. Therefore, circulating IgA is a well-established adverse prognostic indicator in PCs^59,60^. It was reported that the checkpoint molecules were also expressed in the micro-anatomical structures of TLSs. The study of B cell subsets in EAC showed that the high expression of programmed death ligand 1 (PD-L1) or impaired HLA-I expression decreased infiltration of B cells in the tumor tissue^61^.

Furthermore, García-Hernández et al.^62^ observed that CD3^+^Foxp3^+^ and CD3^+^T-bet^+^ T cells accumulated in the T cell areas of TLSs from prostate carcinoma patients. The increased accumulation of Tregs in TLSs correlated with cancer progression, while dramatic enrichment of CD3^+^T-bet^+^T cells inside TLSs coincided with cancer regression. It was also reported that a low CD8/foxp3 ratio in TLSs predicted impaired prognoss in colorectal cancer patients with pulmonary metastasectomies^63^. Moreover, researchers investigated the immune cell subsets and gene expression profiles both in the intratumor and peritumor TLSs of pancreatic ductal carcinomas. The results showed that the tumor-infiltrating foxp3^+^ regulatory T cells and CD163^+^ macrophages (M2) tended to accumulate in the peritumoral TLS. Although Th1-associated genes (*IFNg*, *TBX21*, *IL12B*), Th17-associated genes (*IL17A*, *RORgt*, *IL23R*), inflammation-related genes (*IL6* and *TNFA*), Th2-associated genes (*IL4*, *IL13*, and *GATA3*), and immune suppression related genes (*IL10* and *TGFb1*) were also expressed in TLSs. The upregulation of IL-6 in the presence of TGF-β accelerated the differentiation of Th17 and inhibited the induction of Tregs^64^. A study reported that Tregs accumulated in tumor-associated TLSs and had an activated phenotype with highly expressed activation markers CD44, CD69, and CD39. Tregs depletion increased CD4^+^ and CD8^+^ T cell infiltrations and antitumor responses in lung tumor-bearing mice^65^. Another study assessed the influence of different subsets of TLSs on the prognoses of patients with curatively resected stage II/III colorectal cancers. The results showed that GATA3^+^ Th2 cells and CD68^+^macrophages significantly increased in the recurrence group^66^. The study of soft-tissue sarcomas showed an immunosuppressive microenvironment dominated by CD163^+^ macrophages (M2), which could be overcome with activation of T cells that trafficked into the TME^61^.

Because Tfh is critical for B cell activation and maturation, the state of Tfh directly affects the function of B cells. TIM-3^+^ PD-1^+^ Tfh is significantly more enriched in tumor-infiltrating lymphocytes of ovarian cancer patients than in peripheral blood. This subset of Tfh showed significantly lower levels of IL-21 secretion, lower proliferation, and marked impairment for inducing IgM, IgG, and IgA secretions from B cells^67^. The T follicular regulatory (Tfr) cell is a recently discovered subset of Tregs^68^. The main function of Tfr cells is thought to be inhibition of B cell proliferation and Ig production. A study reported the Tfr-like subsets were characterized with intermediate levels of both Foxp3 and Bcl6, while Tregs levels were as high in Foxp3 and low in Bcl6. The frequencies of Tfr-like cells were significantly more elevated in breast cancer (BC) patients than in noncancer controls. Tfr-like cells increased the production of IL-10 in BC patients, both by directly producing IL-10 and by increasing IL-10 production from B cells. Moreover, the presence of Tfr-like cells significantly reduced Ig production in Tfh-B cell co-cultures^69^. Another study reported that Tfr was found in resected tumor samples of ovarian cancers and could significantly suppress the activation of CD8 T cells, in a manner dependent on IL-10 and TGF-β^70^.

## TLS induction of reprogramming of the TME suggests novel antitumor directions

Strategies to reprogram the TME by establishing TLSs are being explored to improve cancer immunotherapy. It has been reported that immune checkpoint inhibitors (ICIs) induced TLS formation and had anti-tumor functions in the TME. A post-treatment examination of tumor resections from 20 patients with NSCLC who were treated in a phase II clinical trial of neoadjuvant nivolumab (anti-PD-1) showed that the regression bed was characterized by dense TLSs, dense TILs, and plasma cell infiltrates, along with features of cell death (including cholesterol clefts and interstitial foamy macrophages), and tissue repairs/wound healing, such as neovascularization and newly proliferative fibrosis. All these features were absent or rarely present in specimens from non-responding patients^71^.

In addition, a study^72^ reported the presence of massive intratumor TLSs containing both lymphocytes (including CD3^+^T, CD8^+^T, and CD20^+^B cells) and antigen presenting cells (DC-LAMP) in 11 adenomatous polyposis coli (APC) germline mutation hepatoblastoma patients who received cisplatin-based neoadjuvant chemotherapy. In contrast, this feature was only retrieved in a minority of non-*APC* hepatoblastoma cases^72^. Although this was a small sample retrospective study, it also suggested that APC inactivation combined with cisplatin might be able to promote TLS formation through inducing the immunogenical cell death of tumor cells.

Moreover, tumor vaccines have been widely studied and considered to be one of the effective ways to induce TLS formation. The vaccine-targeted DC using adjuvant vector cells engineered from a-GalCer-loaded CD1d and transferred by tumor antigen mRNAs stimulated the development of TLSs and the differentiation of long-term memory T cells. When combined with PD-1 blockers, the vaccine triggered regression of poorly immunogenic tumor cells that did not respond to PD-1 blockade alone and expanded antigen-specific CD8^+^T cell clones in the tumor^73^. Nano-sapper, which consists of a core that is co-loaded with anti-fibrotic phosphate-modified α-mangostin and a plasmid encoding immune enhanced cytokine-a, a member of the tumor necrosis factor superfamily LIGHT (tumor necrosis factor superfamily 14, TNFSF14, CD258), was used to treat pancreatic ductal adenocarcinoma (PDAC)-bearing mice. It reduced the physical obstacles of cancer-associated fibroblasts in the TME, and it induced intratumoral TLSs. Both CCL21 and CXCL13 were significantly increased in the nano-sapper treatment group. Furthermore, the combination of nano-sapper and α-PD-1 achieved better tumor inhibition^74^. LIGHT-VTP, which was developed from fusion compounds composed of mouse LIGHT protein and a carboxy-terminal vascular targeting peptide (VTP), was used in tumor-bearing mice to evaluate its antitumor effects. The results showed a dual ability to modulate the normalization of tumor blood vessels in the PDAC mice model and induce TLSs that induced protective antitumor immunity. LIGHT-VTP, in combination with CTLA-4 and PD-1 inhibition, generated a large number of intratumoral effectors and memory T cells with resulting survival benefits, while the addition of antitumor vaccination achieved maximal therapeutic efficacy^75^. In a melanoma mice model, it was reported that an intratumorally-delivered interferon gene (STING) agonist ADU S-100 (5 µg/mouse) increased the production of antiangiogenic factors including TNFSF15 and CXCL10, and TLS-inducing factors including CCL19, CCL21, LTa, LTb, and LIGHT. Moreover, intratumor STING activation promoted tumor vascular normalization, increased the density of Lyve-1^+^lymphatic endothelial cells, enhanced tumor infiltration of CD8^+^T cells and CD11c^+^ DCs, promoted the local TLS neogenesis, and slowed melanoma growth^76^.

A clinical trial was conducted to evaluate the neoadjuvant effects of TME in PDAC patients using an irradiated granulocyte macrophage colony-stimulating factor-secreting, allogeneic PDAC vaccine (GVAX) in combination with low dose cyclophosphamide (Cy) to deplete Tregs. Thirty-three of 39 patients showed the vaccine-induced neogenesis of intratumor TLSs upon examination of the resected PDAC tissues 2 weeks after treatment, indicating that the immunotherapy converted a “non-immunogenic” neoplasm into an “immunogenic” neoplasm through neogenesis of TLSs. Furthermore, post-GVAX T cell infiltration and TLS analyses showed evidence of early T cell activation and upregulation of the PD-1/PD-L1 T cell suppressive pathway, which indicated the possibility of using combination therapy with checkpoint inhibitors^77^. Another clinical trial evaluated the antitumor effects of vaccination with multiple melanoma peptides in adjuvants comprising incomplete freund’s adjuvant (IFA) and/or the TLR3-agonist pICLC. After repeated vaccination with peptides in IFA, the vaccine site microenvironment showed dramatically enhanced T-bet and decreased GATA3, with high expressions of IFNγ and STAT1, and reduced arginase-1 expression and enhanced expressions of chemokines associated with TLS formation^78^. An intramuscular therapeutic vaccination targeting HPV16 E6/E7 antigens also induced a robust tissue-localized effector immune response in 12 CIN2/3 patients^79^.

High endothelial venules (HEVs) are structurally and functionally distinct postcapillary venules that mediate the adhesion, and transendothelial migration of circulating T- and B-cells to secondary lymphoid organs and TLSs in peripheral tissues suffering from chronic inflammatory stimulation^80^. HEVs are considered to be key factors in the formation of TLSs. A previous study verified that MECA79-positive HEV density in pre-neoadjuvant chemotherapy biopsies was an objective and quantitative marker of TLSs of triple-negative breast cancers^81^. Therefore, attempts to activate the anti-tumor immune response through HEV induction were made. In a preclinical study, the combination of anti-VEGFR2 and anti-PD-L1 antibodies promoted tumor cell destruction by inducing HEV formation and facilitating CTL infiltration and activity in PyMT (polyoma middle T oncoprotein) breast cancer and RT2-PNET (Rip1-Tag2 pancreatic neuroendocrine tumors)-bearing mice. It was reported that anti-VEGFR2 therapy up-regulated the PD-L1 expression in tumors by increasing the percentages of IFN-γ^+^CD8^+^, IFN-γ^+^CD4^+^, and granzyme B^+^(GzB^+^) CD8^+^T cells. The subsequent PD-L1 blockade enhanced vessel normalization, HEV induction, lymphocyte infiltration, and activation^82^.

## Conclusions

In conclusion, the TLS represents a local induction and maintenance of the antitumor immune response in cancer tissues. In functional regions, mature TLSs include not only T cell regions that exert cellular immune responses, but also B cell regions that produce humoral immune responses. Based on these functions, mature TLSs induce a systemic immune response, which is related to good prognoses in cancer patients. Studies has been conducted to explore the strategies, including chemotherapy, vaccine and immunostimulants, which can play a more effective and systematic anti-tumor effect through inducing TLS formation. Because recent studies reported that there were some immunosuppressive factors, such as Bregs and Tregs, which affected the antitumor functions of TLSs, appropriate therapeutic strategies to induce the formation and maturation of TLSs, and at the same time inhibit these immunosuppressive factors, might be a new direction to improve responses to cancer immunotherapy.
